# Family caregivers’ perceptions of the quality of primary healthcare services for people with disabilities: a cross-sectional study

**DOI:** 10.1590/1806-9282.20240778

**Published:** 2024-09-16

**Authors:** Luiza de Sousa Silva, André Pontes-Silva, Eloise Schott, Francisco Winter dos Santos Figueiredo, Leidiene Ferreira Santos, Ladislau Ribeiro do Nascimento, Fernando Rodrigues Peixoto Quaresma

**Affiliations:** 1 Universidade Federal do Tocantins, Postgraduate Program in Teaching in Science and Health – Palmas (TO), Brazil.; 2 Universidade Federal de São Carlos, Department of Physical Therapy, Postgraduate Program in Physical Therapy – São Carlos (SP), Brazil.

**Keywords:** Primary healthcare, Public health, Disabled persons

## Abstract

**BACKGROUND:**

How do caregivers of people with disabilities perceive the quality of health services in primary healthcare?

**OBJECTIVE:**

The objective of this study was to show the quality of health services for people with disabilities in primary healthcare, based on the perceptions of family caregivers.

**METHODS:**

This is a cross-sectional study. During data collection, 49 family caregivers who use the center were interviewed. The assessment instruments used were the Socio-Economic and Demographic Questionnaire and the PCATool-Brasil (Primary Care Assessment Tool), in the reduced adult and child versions, to assess the level of essential and derived characteristics of primary healthcare.

**RESULTS:**

Women were the main caregivers (40; 82%), and the main disability was mental (28; 58%). The highest scores were observed in affiliation (100%), utilization (73.4%), and information system (83.7%). The lowest scores were found in longitudinal (26.5%), integration of care, available services, services provided (28.6%), and derived scores (28.6–22.4%) related to family guidance and community guidance. The population showed a low orientation toward primary healthcare, with a high total score (22.4%). The economic situation showed a positive association (p=0.017).

**CONCLUSION:**

According to the characteristics of primary healthcare, care is fragmented and disjointed and does not meet the needs of people with disabilities and their caregivers.

## INTRODUCTION

Disabled people have specific health needs and therefore have a greater need for health services due to the associated conditions and the variety of care required for their overall well-being. In this context, they naturally face barriers to access and have poorer access to health services^1^. There are at least 1 billion people with disabilities worldwide, representing 15% of the world's population^1^. The United Nations Fund's State of the World's Children report (2021) points out that the global estimate of the number of children with disabilities is higher than previous estimates, estimated at almost 240 million^2^.

Primary healthcare plays a central and structuring role in health systems, coordinated by the health network. For Mendes (2011, p. 91), "the quality of primary healthcare will only exist if it fulfills its essential functions, the function of solvability, inherent to the level of primary care, means that it must be soluble, capable of solving more than 85% of the problems of its population"^3^. Despite its great potential, it still has weaknesses, as Paim et al.^4^ reaffirm that "despite a growing awareness of the importance of quality healthcare in Brazil, much progress is still needed to ensure consistently high standards."

In the context of primary healthcare, people with disabilities face barriers caused by a number of factors: discrimination, physical inaccessibility, inaccessibility and unavailability of information, and lack of knowledge on the part of professionals about disabilities, which contribute to inequalities, whether in health status or access to care, healthcare, which is an essential measure for complete and effective care^1^. As such, the study aims to show the quality of health services for people with disabilities in primary healthcare, based on the perceptions of family caregivers.

## METHODS

### Design

This article is part of a larger study on the "Challenges of Including People with Disabilities in Support Networks: Family Support Perspectives." It is a cross-sectional study conducted according to the guidelines of the STROBE statement. The exposure variables were socioeconomic and demographic characteristics, and the outcome variable was the quality of primary healthcare provided to people with disabilities.

### Setting

The study was conducted in the city of Palmas, Brazil, at the Centro de Atendimento Educacional Especializado Marcia Dias Costa Nunes (CAEE), which provides educational assistance to students with physical and mental disabilities. The center is part of the state education network. Studies evaluating primary healthcare often use the work setting itself (health services) or its surroundings to conduct research. To reduce interviewer bias, the interviews were conducted from an educational perspective. It took place from October to December 2022. Data collection took place at the educational unit itself and remotely.

### Participants

The participating population consisted of family caregivers belonging to the CAEE. Non-probabilistic convenience sampling was used. Inclusion criteria were based on being a career in the CAEE, being able to respond, and being a user of the service being evaluated. Failure to make an appointment and/or telephone contact for an interview after three attempts was considered a loss, as was a lack of acceptance and being a formal carer.

### Variables

The socioeconomic, demographic, and other characteristics of family caregivers were based on information such as gender, education, Brazilian economic level, degree of affiliation, time spent caring for people with disabilities, type of disability, and use of health services.

The PCATool-Brazil short version (child and adult) was used to assess the extent of essential and derived attributes of primary healthcare provided by family caregivers. This tool was originally developed by Bárbara Starfield's team and her colleagues at the Johns Hopkins Population Policy Center. In relation to the attributes measured by the PCATool—Brazil set of primary healthcare instruments, the socioeconomic and demographic situation of family caregivers and their perceptions of health services were collected through a structured questionnaire prepared by the researchers, in accordance with the Brazilian criteria of the Associação Brasileira de Empresas de Pesquisa (ABEP)^5^.

The reduced version of the Primary Healthcare Assessment Instrument for Child Patients, whose validity and reliability measures are known for Brazil, consists of 30 items divided into 10 components related to the characteristics of primary healthcare. The items that make up the instrument have responses on a Likert scale ("4=definitely yes," "3=probably yes," "2=probably no," and "1=definitely no") with the addition of the option "9=I do not know/cannot remember." From these responses, it is possible to calculate a score for each primary healthcare attribute, the essential score, and the overall score. In this way, the presence and extent of each primary healthcare attribute can be independently assessed, as can the essential attributes (essential score) and the degree of general orientation of primary healthcare services (general score).

### Sample size

According to the institution's records, there were 127 people with disabilities being cared for by family caregivers (n=122; 96.06%), all of whom were invited to participate voluntarily, in person and/or by telephone.

### Statistical methods

Quantitative variables were described using absolute and relative frequencies, mean (standard deviation), and minimum and maximum values. Fisher's exact test was used to analyze the association of socio-demographic variables, characteristics of the person with a disability, perception of use, and access to health services with the high general primary healthcare score. To classify the high score, the PCATool-Brazil guide was used, a reduced version that classifies this cut-off point (≥6.6) as a high score since it is the value that reflects the minimum presence of services related to primary healthcare. The significance level was 5%. The STATA software (StataCorp, LLC) version 16.0 was used.

## RESULTS

### Participants

According to the data provided by the institution, 127 people with disabilities were enrolled and cared for by 122 family members (difference of more than one enrolled child), who met the eligibility criteria. A total of 73 people were excluded. Losses were considered due to absence after three attempts to contact for collection (n=67; 54.92%), no interest (n=05; 4.10%), and no criteria (n=1; 0.82%). Respondents were successfully contacted (n=49; 40.16%).

### Descriptive data

The main characteristics of caregivers in this study were mothers (n=40; 82%), duration of caregiving (n=31; 63%), and the most commonly reported type of disability, intellectual disability (n=28; 58%). Table 1 summarizes the main characteristics of caregivers and people with disabilities. Table 2 shows factors associated with poor indication for primary care attributes.

**Table 1:** Characteristics of caregivers and disabled people.

Variable		n (%)
Degree of relationship		
	Mother	40 (82)
	Other	1 (2)
	Father	6 (12)
	Disabled people	1 (2)
	Responsible	1 (2)
Care period (years)		
	<1	3 (6)
	1–2	6 (12)
	3–4	8 (16)
	>5	31 (63)
	n/a	1 (2)
Type of disability		
	Hearing disability	4 (8)
	Intellectual disability	28 (58)
	Multiple disability	25 (26)
	Visual disability	4 (8)
Using the health service (years)		
	0.6–1	8 (16)
	~5	21 (43)
	~10	11 (22)
	~15	9 (18)

**Table 2:** Factors associated with poor indication for primary care attributes.

Variable		Low primary healthcare score (%)	High primary healthcare score (%)	p-value
Sex				0.65
	Female	33 (79)	9 (21)	
	Male	5 (71)	2 (29)	
Education				0.56
	Complete primary	3 (100)	0 (0)	
	Incomplete primary	1 (100)	0 (0)	
	Complete secondary	17 (81)	4 (19)	
	Incomplete secondary	4 (100)	0 (0)	
	Complete undergraduate	7 (58%)	5 (42)	
	Incomplete undergraduate	6 (75)	2 (25)	
Brazilian economic level (R$)				0.017
	Upper class upper (>9,920)	0 (0)	2 (100)	
	Upper middle class (4,076)	3 (43)	4 (57)	
	Average middle class (2,564)	15 (83)	3 (17)	
	Lower middle class (1,764)	14 (88)	2 (13)	
	Vulnerable (1,164)	5 (100)	0 (0)	
	Poor (648)	1 (100)	0 (0)	
Disability (type)				0.17
	Hearing disability	2 (50)	2 (50)	
	Intellectual disability	23 (82)	5 (18)	
	Multiple disability	10 (83)	2 (17)	
	Visual disability	2 (50)	2 (50)	
Using the health service (years)				0.37
	~5	18 (86)	3 (14)	
	~10	9 (82)	2 (18)	
	~15	5 (56)	4 (44)	
	0.6–1	6 (75)	2 (25)	

### Outcome data

**Figure 1:**
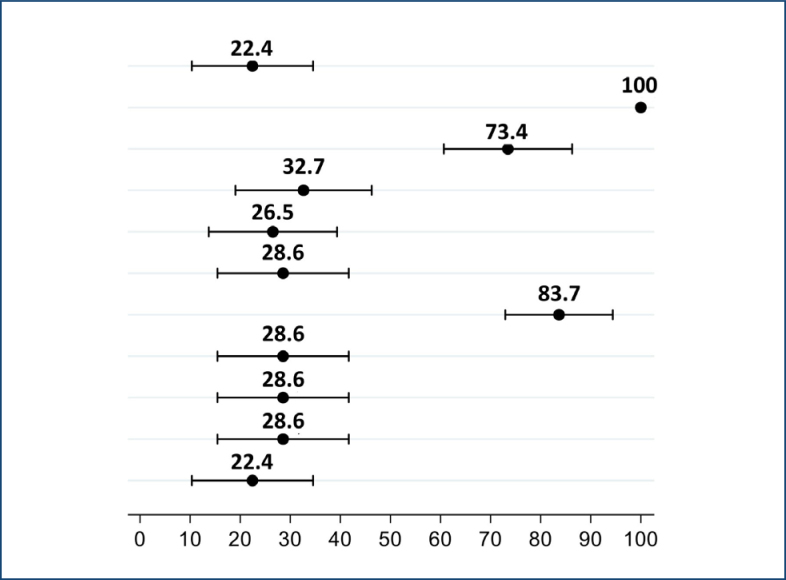
Rate of high scores (cut-off point ≥6.6) on attributes and overall primary healthcare score.

Regarding the quality of primary healthcare, Figure 1 shows the rate of high scores (cut-off point ≥ 6.6) for attributes and the overall primary healthcare score, which shows average scores for attributes with a 95% confidence interval, based on the experiences of family caregivers and people with disabilities using primary healthcare services.

The average overall score was 22.4%, below the cut-off for good general primary care, which was set at ≥ 6.6. If we break this analysis down by attribute, we can see the reasons for these scores. Overall, the contributions of "membership," "utilization," and "information systems" helped to improve the score, while "accessibility," "longitudinally," "integration of care," "services provided," and "services available" were negative.

The highest scores for strong primary care leadership were for affiliation [100%], utilization [73.4%], and information system [83.7%]. These attributes received the highest scores. From another point of view, the worst scores, representing a weak orientation of primary healthcare, were found in longitudinally [26.5%], integration of care, available services, services provided [28.6%], and the derived score related to family orientation and guidance community [28.6 to 22.4%].

## DISCUSSION

### Key results and interpretation

The quality of health services shows a low orientation toward primary healthcare, which is associated with a population with a lower income, according to family caregivers. The existence of some factors associated with a low orientation toward the attributes of primary healthcare can be seen in the prevalence of women as the main caregivers of children with disabilities and families with lower incomes.

The results corroborate other studies that have presented the profile of low-income mothers as the main caregivers of children with disabilities and a lifestyle characterized by fatigue, the cancellation of their own lives, professional decline, and stress, among others^6^. In spite of current social changes, our culture still makes women responsible for caring. Changes in family configuration, with the presence of other members, can provide more time for self-care^7^.

The prevalence of intellectual disability in the study shows a different scenario from the Brazilian ranking, preceded by visual (18.6%), motor (7%), and hearing (5.10%) disabilities^8^. There are authors^7^ who point out that intellectual disability poses greater challenges for education and work and that the people who make up this group will face important barriers, from few opportunities as they age, low social participation, prejudice, and marginalization, to their inclusion/participation.

The scores observed for the degree of affiliation and use, i.e., recognition and use of the health service, show that those responsible identify primary healthcare as a reference service for disabled people. The results found show low scores in the accessibility sub-item, which expresses a disadvantaged situation in health services, which in some way directly implies the implementation of health policies, considering that accessibility consists of this first movement of evaluating the gateway of health systems^9^.

A study evaluating access to primary healthcare for children and adolescents showed that many emergencies could have been resolved in primary care and that many users prefer to go directly to emergency services, often because they value higher-density services technological, distorting the concept of complexity, where primary healthcare is characterized as "basic care," which brings with it the notion of "elementary" or "less complex"^10^.

Caregivers of children with disabilities identify primary healthcare as a gateway, which suggests some explanations as the only alternative for the low-income profile found, demonstrating the strategic importance of the Unified Health System^4^, to reduce social inequalities, especially the right to health. The search for universal access, initially guaranteed by the Brazilian Federal Constitution, ensures equal access to health services for people with disabilities. However, without considering the quality of services, it may not achieve the desired effectiveness and have a direct impact on those who use health services^11^.

In other countries that have universalism as a public policy, existing inequalities are one of the most important challenges to be faced. China has adopted strategies to contribute to the Sustainable Development Goals (SDGs), defined in the 2030 Agenda, in particular SDG 3, which defines quality health, through the construction of an integrated and cooperative primary healthcare system that supports employees fully and is responsible for their performance. However, both systems still face challenges in their structural characteristics. India's path prioritized investments in the budgetary allocation of other public health policies, especially for low-income families, the improvement of primary healthcare, and the expansion of the health workforce^12^.

The results found reflect, in some way, the complexity of the health system and the challenges of inclusion and access for people with disabilities. As it is a condition that results in long-term disability in terms of the barriers faced, it has a direct impact on social participation under conditions of equality^13^. According to Starfield^9^, longitudinally is a characteristic that refers to the continuity of care, which is essentially the relationship established over time between individuals and a professional and/or healthcare team, and presupposes the existence of a regular source of care and its use over time, regardless of the presence of pathology.

The positive evaluation of this attribute indicates loyalty to the services, which was not observed in this study. Continuity of care is directly related to the receipt of information, trust, and security in the care pathway, as well as a relationship of trust with the professional, which are factors that anchor continuity^14^. In terms of lifelong care, longitudinal care can be disrupted by the turnover of professionals who make up primary healthcare, which means that long-term follow-up does not take place, and this scenario represents a further obstacle for people with disabilities. A study of the Brazilian health system^4^ showed progress in the way primary healthcare professionals are recruited, and although the number of temporary jobs has decreased, the primary healthcare workforce still has high turnover.

When dealing with people with disabilities, it is important to stress the importance of professionals getting to know their patients better, their needs, and their specificities. Due to the longevity of care, if it is not effective, it can have negative effects, interfere with the permanence of the service, planning, and adequate intervention of care, and lead to the search for specific assistance at other points of care. Care coordination received an unsatisfactory score for care integration and a satisfactory score for the information system. In line with another similar study, there is a weakness in the coordination of care as a whole, in contrast to the proposal of primary healthcare as an organizer of healthcare, with compromised management and continuity of care^15^.

It is important to emphasize that this is a descriptive study, and there is little contribution to factors that may be related to the perception of the quality of services provided. In addition, the sample size is limited, and we suggest that the conclusions presented may not be extrapolated to other areas.

## CONCLUSION

The importance of continuing professional development, the visibility of user and professional perspectives, effective communication, investment in the structuring and organization of primary services, guaranteed funding, and a specialized network linked to primary healthcare units are seen to overcome the challenges faced by family caregivers and people with disabilities through the joint efforts of managers, individuals, and society.

## ETHICS APPROVAL

This study was approved by the Research Ethics Committee of the Universidade Federal do Tocantins (report number: 63158622.0.0000.5516).
